# Autophagy mediates an amplification loop during ferroptosis

**DOI:** 10.1038/s41419-023-05978-8

**Published:** 2023-07-25

**Authors:** Seunghee Lee, Narae Hwang, Byeong Geun Seok, Sangguk Lee, Seon-Jin Lee, Su Wol Chung

**Affiliations:** 1 Department of Biological Sciences, College of Natural Sciences, University of Ulsan, 93 Daehak-ro, Nam-gu, Ulsan, 44610 South Korea; 2 Division of Pulmonary, Allergy, and Critical Care Medicine, Department of Medicine, VA Palo Alto Health Care System and Stanford University School of Medicine, Palo Alto, CA 94305 USA; 3 Division of Pulmonary and Critical Care Medicine, Department of Medicine, Brigham and Women’s Hospital and Harvard Medical School, Boston, MA 02115 USA; 4 Environmental Disease Research Center, Korea Research Institute of Bioscience and Biotechnology, Yuseong-gu, Daejeon, 34141 South Korea; 5 Basic-Clinical Convergence Research Institute, University of Ulsan, Ulsan, 44610 South Korea

**Keywords:** Autophagy, Stress signalling, Cancer, Cell death, Mitophagy

## Abstract

Ferroptosis, a programmed cell death, has been identified and associated with cancer and various other diseases. Ferroptosis is defined as a reactive oxygen species (ROS)-dependent cell death related to iron accumulation and lipid peroxidation, which is different from apoptosis, necrosis, autophagy, and other forms of cell death. However, accumulating evidence has revealed a link between autophagy and ferroptosis at the molecular level and has suggested that autophagy is involved in regulating the accumulation of iron-dependent lipid peroxidation and ROS during ferroptosis. Understanding the roles and pathophysiological processes of autophagy during ferroptosis may provide effective strategies for the treatment of ferroptosis-related diseases. In this review, we summarize the current knowledge regarding the regulatory mechanisms underlying ferroptosis, including iron and lipid metabolism, and its association with the autophagy pathway. In addition, we discuss the contribution of autophagy to ferroptosis and elucidate the role of autophagy as a ferroptosis enhancer during ROS-dependent ferroptosis.

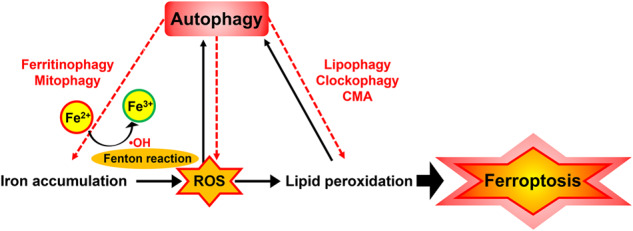

## Facts

1. Ferroptosis is a non-apoptotic form of regulated cell death.
2. Ferroptosis is dependent on iron accumulation and ROS generation.
3. Oxidative stress contributes to ferroptosis.

## Questions

1. Does autophagy contribute to ferroptosis?
2. What are the molecular mechanisms underlying the regulation of ferroptosis by autophagy?
3. How do ROS and iron accumulation increase ferroptosis on a molecular level?

## Ferroptosis

Cell death is critical in the development of multiple human diseases and is closely linked to biological growth. Ferroptosis is a new type of cell death discovered in the past decade. Initially, ferroptosis has been discovered as a novel iron-dependent form of non-apoptotic regulated cell death. Ferroptosis is associated with the accumulation of iron, ROS, lipid peroxidation, and insufficient capacity to eliminate lipid peroxidation [1–4]. Moreover, ferroptosis is closely associated with the pathophysiology of several diseases including tumors [2], degenerative diseases [5], ischemia-reperfusion injury [6], kidney injury [1], and blood disorder [7], and has a tumor suppressor function that can be used in anti-cancer [2, 8]. Particularly, studies have shown that targeting ferroptosis could be a potential strategy in different cancer cell types. A study has shown that AML cells with mutations in the IDH1 or IDH2 genes are particularly sensitive to ferroptosis-inducing agents and that this sensitivity is mediated by the accumulation of the oncometabolite 2-hydroxyglutarate (2-HG) [9]. In addition, inhibiting ferroptosis in liver cancer cells promoted tumor growth and metastasis, while inducing ferroptosis inhibited tumor growth and reduced metastasis [2]. Another study reported that ferroptosis is involved in the regulation of tumor immune response in melanoma, a type of skin cancer [10].

Ferroptosis pharmacological modulation has emerged as a research focus for the treatment and prognosis of related diseases. While ferroptosis has been shown to have anti-tumor effects in some contexts, it can also promote tumor growth and survival in other contexts. As an anti-tumor effects, a classical ferroptosis inducer is associated with the System Xc-/glutathione (GSH)/glutathione peroxidase 4 (GPX4) axis, which plays a central role in the regulation of the antioxidant system and lipid peroxidation [11, 12]. In addition to oncogenic RAS (Rat sarcoma virus oncogene)-selective lethal small molecules, erastin, RAS-selective-lethal compound 3 (RSL3), and other chemical compounds, such as sulfasalazine, sorafenib, artemisinin, and 1, 2-dioxolane (FINO2), have been confirmed to induce ferroptosis [11, 13]. For example, in certain subtypes of cancer, such as glioblastoma and acute myeloid leukemia (AML), the induction of ferroptosis has been shown to have anti-tumor effects [14].

As opposite effects on tumor cell survival and growth, ferroptosis inhibitors may be used as treatment strategies in certain types of diseases with enhanced ferroptosis. Ferroptosis has been shown to promote tumor growth and survival in cancers such as pancreatic cancer [15]. In these cases, the use of ferroptosis inhibitors could potentially be a viable adjuvant treatment strategy to inhibit tumor growth. Targeting the key enzymes and molecules involved in the ferroptosis process is one potential approach to developing ferroptosis inhibitors for cancer treatment. For example, inhibitors of the lipid peroxidation enzyme, 15-lipoxygenase (15-LOX), have been shown to inhibit ferroptosis in cancer cells, and may have therapeutic potential for certain types of cancer [16].

Initially, the oncogenic RAS-selective lethal small molecule erastin-induced ferroptosis was described as autophagy-independent cell death in fibrosarcoma HT-1080 cells [17]. However, studies have demonstrated that autophagy plays a role in regulating iron content, ROS congestion, and lipid peroxidation during ferroptotic cell death [18, 19]. Thus, understanding autophagy and ferroptosis regulation could help treat tumors, inflammatory diseases, and fibrosis. Despite increasing evidence, the role and pathophysiology of autophagy during ferroptosis remain unclear [20]. In this review, we provide an overview of autophagy and ferroptosis and a comprehensive review of the molecular mechanisms of autophagy-dependent ferroptosis and its regulation.

### Mechanisms of iron accumulation during ferroptosis

Iron is an essential component of biological systems and exists in two biologically relevant oxidation states, ferric iron (Fe^3+^) and ferrous iron (Fe^2+^), and functions as a cofactor for several proteins and enzymes that can readily undergo redox cycling. Specifically, excessive amounts of iron initiate several cytotoxic mechanisms that disrupt redox homeostasis and cause cell death, including ferroptosis [21].

**Fig. 1:**
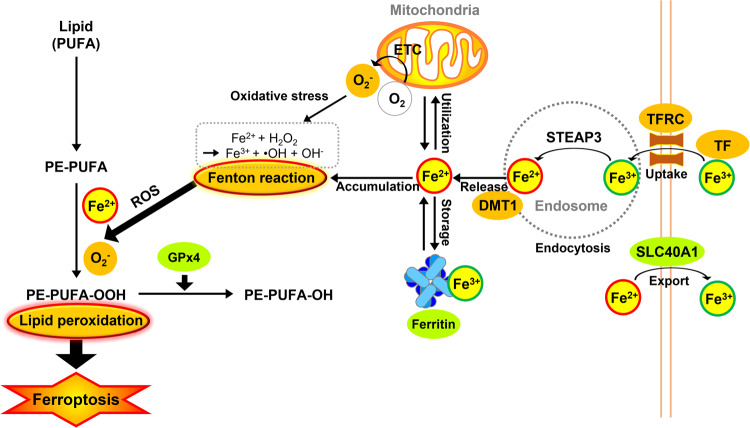
Molecular metabolism of iron in ferroptosis. During the process of ferroptosis induction, the increase of cytoplasmic instability of iron is observed. The overall process of iron metabolism, including iron uptake, storage, utilization, and efflux is involved in the regulation of ferroptosis. Fe^3+^ forms a complex with circulating transferrin (TF) and then, a Fe^3+^-TF complex binds to the transferrin receptor (TFR) and is transported inside the cell to the endosome. After endocytosis, Fe^3+^ is detached from TF and converted to Fe^2+^ via redox reaction by STEAP3 and then exported into the cytosol by DMT-1. Unbound Fe^2+^ in the cytosol can be transported to the mitochondrion and labile iron are stored by ferritin. Excess Fe^2+^ is exported out of the cell through SLC40A1, known as ferroportin-1 maintaining systemic iron homeostasis. Labile iron displays high chemical reactivity and cytotoxic potential. Fe^2+^ plays an important role in the development of ferroptosis via enzymatic lipid peroxidation by iron-containing enzymes, such as ALOXs or nonenzymatic lipid peroxidation by Fenton reaction catalyzing the formation of hydroxyl radicals (OH·) [25, 118].

As iron regulates ROS production and enzyme activity in lipid peroxidation, iron homeostasis maintenance via iron metabolism, including iron uptake, storage, utilization, and export, provides a unified network to determine ferroptosis sensitivity [12]. Most biological iron is derived from nutritional sources and hemoglobin, a red blood cell component, and is directly absorbed into the bloodstream through intestinal mucosal cells, and is partly stored in ferritin [22]. Transferrin (TF), delivered as a glycoprotein, binds to Fe^3+^ and transports it through the blood to various tissues, such as the liver, spleen, and bone marrow. TF-bound ferric iron from the serum is recognized by the transferrin receptor (TFRC/TfR1) followed by internalization of the TF-TFRC complex by receptor-mediated endocytosis (Fig. 1). Increased iron uptake by TFRC overexpression enhances sensitivity to ferroptosis, and TFRC knockdown ameliorates erastin-induced ferroptosis. Thus, TFRC may serve as a biomarker of ferroptosis sensitivity [12, 22].

After uptake by TFRC, endosomal iron is released from TF and is reduced from Fe^3+^ to Fe^2+^ by STEAP3 metalloreductase [23]. Iron is then released from the endosomal compartment into the cytoplasm by the divalent metal transporter DMT1 (DCT1, Nramp2, SLC11A2; solute carrier family 11 member 2). DMT1 (SLC11A2) sensitizes cells to ferroptosis by increasing cytosolic iron levels [24].

Unbound cytosolic Fe^2+^ can be transported to the mitochondria or form a labile iron pool that is stored by ferritin. Fe^2+^ regulates multiple processes, such as oxygen transport and iron-sulfur (Fe-S) assembly in mitochondria [22, 25]. Ferritin takes up labile Fe^2+^ and stores it in a stable unreactive Fe^3+^-oxide/hydroxide form [26]. Ferritin consists of two subunits, heavy chain 1 (FTH1) and light chain (FTL). Ferritin degradation can occur via ferritinophagy, a selective autophagy of ferritin, and is a prerequisite for iron release. Therefore, reducing iron storage via the knockdown of ferritin protein or induction of ferritinophagy increases ferroptosis [27]. The efflux of intracellular Fe^2+^ across the cell membrane requires the iron efflux pump ferroportin-1 (SLC40A1). Iron efflux by SLC40A1 alleviates iron overload and decreases ferroptosis [28].

Excess Fe^2+^ accumulates and reacts with hydrogen peroxide (H_2_O_2_) via the Fenton reaction, a metal-catalyzed reduction of hydrogen peroxide, to form highly toxic hydroxyl free radicals (•OH). Fe^2+^ acts as a catalyst, facilitating the conversion of H_2_O_2_ into •OH and hydroxide ions (OH^-^) [26, 29, 30]. The generated •OH can bring about damage to DNA, proteins, lipid membranes, and other biomolecules [26, 31].

### Molecular mechanisms of lipid peroxidation during ferroptosis

Excessive lipid peroxidation leads to cell death through ferroptosis. Lipid peroxidation is the process to generate lipid hydroperoxides (LOOHs) through which intermediate of peroxyl radicals formatted by ROS combines with lipids (ex-PUFA/PL;phospholipid) [32]. During ferroptosis, Acyl-CoA synthetase long-chain family member 4 (ACSL4) and lysophosphatidylcholine acyltransferase 3 (LPCAT3) take charge of most PUFAs production and promote the esterification of PUFA (AA and AdA) into phosphatidylethanolamine (PE) [33, 34]. Thus, ACSL4 inhibitors such as rosiglitazone, pioglitazone, and troglitazone decrease ferroptosis by regulating cellular sensitivity [32, 35].

**Fig. 2:**
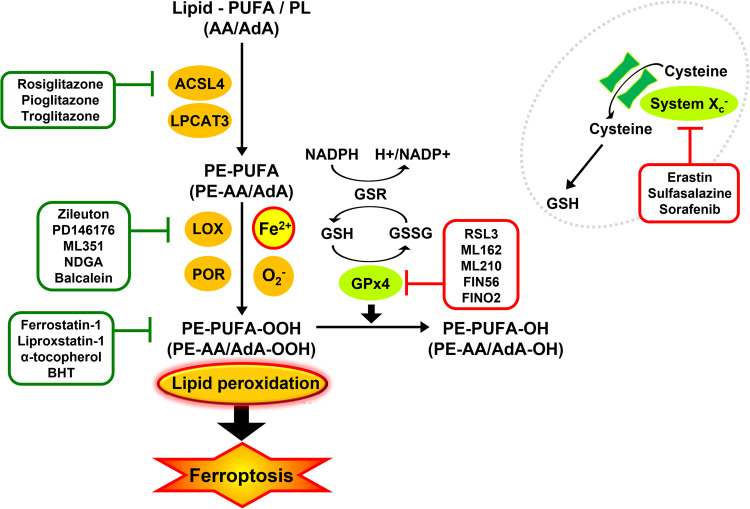
Summary of lipid peroxidation mechanisms and signaling pathway. The pathway regulating sensitivity during the generation of lipid peroxidation. PUFA is a double-sided, and its peroxidation contributes to cell damage. The production of PUFA derivatives requires the activation of the ACSL4-LPCAT3 enzymes pathway. PE-PUFA oxidation could occur either enzymatically via the action of Lox, or non-enzymatically via autoxidation to form PE-PUFA-OOH, both of which after all trigger ferroptosis. ROS produced by the Fenton reaction directly promotes lipid peroxidation and iron may increase the activity of ALOX, which are iron-containing enzymes. The toxic phospholipid hydroperoxides (R-OOHs) are detoxified to nontoxic phospholipid alcohol (R-OH) by GPX4 by consuming two glutathione (GSH) molecules as an electron donor. The ferroptosis inhibitors are shown in Green line, while the ferroptosis inducers are shown in Red line [48, 52].

Among biological membrane phospholipids, phosphatidylethanolamine (PE) and phosphatidylcholine (PC) including arachidonic acid (AA)- and adrenic acid (AdA) are principal targets for lipid peroxidation [33]. The peroxidation of PE-AA/AdA is enzymatically catalyzed by lipoxygenases (LOXs) and cytochrome P450 oxidoreductase (POR), or non-enzymatically by cellular free iron via the Fenton reaction [33, 36]. LOX inhibitors, such as Zileuton, PD146176, ML351, and NDGA, inhibit ferroptosis (Fig. 2) [37].

Dysregulation of membrane lipid peroxidation is induced by loss of the lipid detoxification enzymatic GPX4, and subsequent accumulation of lipid-based ROS, particularly lipid hydroperoxides [3, 38]. The GSH/GPX4 axis is the most often targeted pathway for causing ferroptosis [32]. GPX4 reduces lipid peroxide (PE-AA/AdA-OOH) to lipid alcohol (PE-AA/AdA-OH) by oxidizing the GSH synthesized from cysteine, thereby defending cells from ferroptosis under normal conditions. Loss of GPX4 activity or depletion of GSH and inhibition of cysteine uptake leads to increased lipid peroxidation and ferroptosis [33]. Thus, GPX4 inhibitors, such as RSL3, ML162, ML210, FIN56, and FINO2 are classic ferroptosis inducers [37]. Furthermore, inhibitors of system Xc− as cystine transporters, such as erastin, sulfasalazine, and sorafenib, trigger ferroptosis [39]. Moreover, lipid peroxide (R-OOH) is suppressed by scavengers of lipid peroxidation involving ferrostatin-1, liproxstatin-1, Vitamin E (α-tocopherol), butylated hydroxytoluene (BHT) (Fig. 2) [40].

## Molecular mechanisms of oxidative stress induction and ferroptosis

Ferroptosis is an iron-dependent oxidative cell death pathway characterized by the oxidative modification of phospholipid membranes via lipid peroxidation caused by ROS from the Fenton reaction [41, 42]. Iron-dependent oxidative stress can damage lipids and exacerbate lipid peroxidation [41, 43, 44]. Extensive oxidation of PUFA-containing phospholipids alters the membrane’s phase structure and permeability, resulting in plasma membrane rupture in response to lipid-ROS accumulation, such as lipid hydroperoxides [45].

Three main sources of lipid peroxidation during ferroptosis are hypothesized: (1) Fenton reaction-induced ROS caused by elevated intracellular iron levels increase iron-dependent oxidative stress and subsequent lipid peroxidation, (2) Mitochondrial iron accumulation and mtROS involved in lipid peroxidation, and (3) Antioxidant defense deficiency leading to lipid peroxidation [37, 42, 46].

**Fig. 3:**
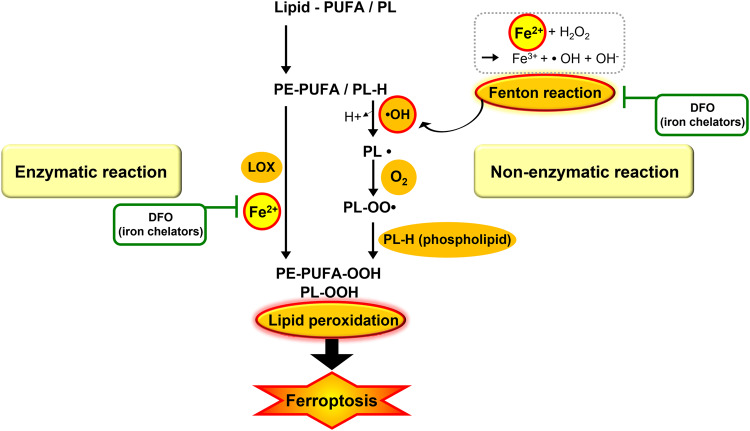
The involvement of iron in enzymatic or non-enzymatic lipid peroxidation. Labile iron has high chemical reactivity and cytotoxic potential. Fenton reaction as an oxidation process between Fe^2+^ and H_2_O_2_, is related to the non-enzymatic lipid peroxidation resulting in forming of a lipid radical (L•) via subtracting hydrogen (H) via providing hydroxyl radicals (OH·) to lipid (L-H). lipid radical (L•) binds to O_2_, thereby forming lipid peroxyl radical (LOO•), subsequently combine with hydrogen (H) exroted from nearby PUFA and form lipid hydroperoxides (LOOH) and a new lipid radical and triggers another oxidation reaction [48]. Phosphatidylethanolamines (PE); phospholipid (PL-H); phospholipid alkoxyl radical (PL-O·); phospholipid peroxyl radical (PL-OO·); phospholipid hydroperoxide (PL-OOH).

Abnormal iron metabolism may induce ferroptosis in at least two ways. First, ROS generation is mediated by iron via the Fenton reaction, and second, the activation of iron-containing enzymes, such as LOX (Fig. 3) [37]. Through the Fenton reaction, free iron reacts with H_2_O_2_, producing hydroxyl radicals, the most reactive radicals. The hydroxyl radical can nonenzymatically mediate the peroxidation of PE-FA. Hydroxyl radicals take hydrogen from PE-PUFAs or phospholipids to form a phospholipid carbon-centered radical. Subsequent oxygen addition to the peroxyl radical yields phospholipid hydroperoxide, which can promote ferroptosis [41, 47].

Furthermore, iron as a cofactor of LOX contributes to enzyme-mediated peroxidation [42, 48]. It should be noted that both cellular lipid hydroperoxides regulated by the LOXs enzyme and autoxidized peroxyl radical-mediated lipid peroxidation may promote ferroptosis initiation. Therefore, accumulated intracellular iron triggers ferroptosis, while iron chelators such as deferoxamine (DFO) reduce free radical production and delay ferroptosis (Fig. 3) [41, 42].

Unbound cytosolic Fe^2+^ can be taken up by mitochondria (Fig. 1), which use a consistent amount of cellular iron as a cofactor for several proteins involved in redox reactions of the respiratory chain [21]. Mitochondria have been proposed to regulate ferroptotic signaling pathways through control of ATP synthesis, ROS production, iron homeostasis, and redox status. Recent studies have demonstrated that several mitochondrial proteins, such as CDGSH iron sulfur domain 1 (CISD1) and Frataxin (FXN), also regulate ferroptotic cell death by mediating iron uptake and lipid peroxidation [21, 49].

GSH, a non-enzymatic antioxidant, and GPX4, a GSH-dependent antioxidant enzyme GSH peroxidase 4, play an important role in protecting cells against various oxidative stress. GPX4 converts GSH into GSSG and reduces cytotoxic lipid peroxides to their corresponding lipid alcohols. Loss of GPX4 activity and GSH depletion cause an imbalance in cellular antioxidant system, leading to the accumulation of lipid peroxides, which is a key feature of ferroptosis (Fig. 2) [2, 50].

## Autophagy in ferroptosis

Autophagy is an evolutionarily conserved self-degradative process to maintain cellular homeostasis, mediated by the autophagosome as a double layer-membrane structure that undergoes maturation by fusing with lysosomes for degradation [51, 52]. Recent studies have suggested that autophagy plays an essential role in ferroptosis [20, 51, 53]. In normal cells, autophagy acts as a protective mechanism against the accumulation of damaged proteins and organelles, which can lead to cancer development. However, in some cases, autophagy can promote cancer cell survival and growth by providing them with the necessary nutrients and energy they require to maintain their metabolic requirements. Several studies have shown that autophagy plays a complex role in different subtypes of cancer. For example, a study found that autophagy promotes the survival of pancreatic cancer cells by protecting them from cell death induced by chemotherapy drugs [54]. Another study discovered that autophagy promotes the survival and metastasis of breast cancer cells by enhancing their ability to invade and migrate to other tissues [55]. Particularly, the relationship between autophagy and ferroptosis in cancer cells is complex and context-dependent. Autophagy can promote ferroptosis in cancer cells by degrading and recycling iron-storage proteins, such as ferritin, which can release iron and promote lipid peroxidation and oxidative stress. A study found that inducing autophagy in cancer cells increased the sensitivity of these cells to ferroptosis-inducing agents, such as erastin and RSL3 [11]. In contrast, autophagy can also inhibit ferroptosis in cancer cells by removing damaged or oxidized lipids that can trigger ferroptosis. A previous study reported that inhibiting autophagy in liver cancer cells increased the accumulation of oxidized lipids and enhanced their resistance to ferroptosis [56]. Therefore, the effect of autophagy on ferroptosis in cancer cells depends on the specific context and conditions of the tumor microenvironment.

There is evidence that certain gene mutations in cancer can affect the sensitivity to ferroptosis. For example, mutations in the Kras oncogene have been shown to promote resistance to ferroptosis in lung cancer cells [57]. Mutations that activate the PI3K/Akt/mTOR pathway, which is involved in cell survival and proliferation, have been associated with resistance to ferroptosis. For example, mutations in the PIK3CA gene, which encodes the p110α subunit of PI3K, have been shown to promote resistance to ferroptosis in breast cancer cells [58]. In contrast, mutations that activate the tumor suppressor gene p53 or its downstream targets, such as the BCL-2 family of proteins, have been associated with enhanced sensitivity to ferroptosis. For example, a previous study discovered that loss of the tumor suppressor gene LKB1, which leads to activation of the AMPK pathway and inhibition of mTOR signaling, enhances sensitivity to ferroptosis in lung cancer cells [59]. The role of autophagy in these cancers appears to be complex and context-dependent. Autophagy can promote either cell survival or cell death in response to ferroptosis-inducing agents, depending on the specific context. For example, a study reported that autophagy inhibition enhanced sensitivity to ferroptosis in pancreatic cancer cells, while a study showed that autophagy inhibition promoted ferroptosis-induced cell death in KRAS-mutant lung cancer cells [60]. Collectively, the interplay between gene mutations, ferroptosis, and autophagy in cancer is complex and requires further investigation.

However, the mechanisms of autophagy-dependent ferroptosis remain unknown, and further investigation of the mediators of autophagy induction during ferroptosis is required [20]. Understanding the relationship between ferroptosis and autophagy will help develop novel treatments for numerous diseases. Here, we outline the key role of the autophagy machinery in the promotion of lipid peroxidation and iron accumulation.

### The role of autophagy in promoting iron accumulation

Previous studies have shown that excessive autophagy and lysosomal activity can promote ferroptotic cell death through iron accumulation. Ferritinophagy, the process of autophagic degradation of the iron-storage protein ferritin, reduces iron storage and promotes cellular iron accumulation through the release of free iron. Thus, ferritinophagy causes oxidative injury via the Fenton reaction and induces ferroptosis. Nuclear receptor coactivator 4 (NCOA4) as a selective cargo receptor responsible for autophagic ferritin degradation binds to ferritin through its C-terminal domain and delivers it to the nascent autophagosome [22, 61, 62].

However, the role of mitophagy, selective autophagy of mitochondria, in ferroptosis is complicated [63, 64]. During the early stages of iron overload, a significant amount of released iron is transported to the mitochondria as a buffer, and mitophagy may segregate iron into the mitophagosomes and decrease the origin of ROS for ferroptosis [65, 66]. However, mitochondrial damage by excessive iron overload induces further mitophagy, providing an additional source of iron for lipid peroxidation [67]. Eventually, extensive mitophagy releases iron, ROS, and peroxidated lipids from mitochondria at toxic levels, thereby activating various ROS-induced cell death pathways, including ferroptosis [37, 68].

Inhibition of the mitochondrial respiratory chain complex I by BAY 87-2243 induces the opening of mPTP and a loss of mitochondrial membrane potential (Δψ), thereby leading to the activation of ferroptosis via ROS increase by stimulated mitophagy [69, 70], while the knockdown of PINK1 inhibits BAY-induced ferroptosis [68]. Although several studies on mitophagy and ferroptosis are ongoing, little is known about how mitophagy-related mitochondrial proteins regulate ferroptosis, so further research is needed [49, 67, 71, 72].

### Molecular mechanisms and the role of autophagy in promoting lipid peroxidation

The consumption of fat and lipids have been shown to impact autophagy and ferroptosis regulation in different diseases, especially in prostate and liver cancer cells [2, 73]. High-fat diets have been shown in their studies to decrease autophagy and increase susceptibility to ferroptosis in some types of cancer cells, which may contribute to cancer development and progression.

Specifically, de novo-synthesized fatty acids are stored in lipid droplets as storage sites for neutral lipids and in phospholipids in the membrane. Lipid droplets (LDs) act as potential ROS scavengers and inhibitors of PUFA oxidation [74–76]. Free fatty acids (FFAs) released by the breakdown of LDs generally serve as fuel for mitochondrial fatty acid β-oxidation for energy production and increase the peroxidation of PUFA and ferroptosis [77]. Therefore, LDs are emerging as modulators of lipid peroxidation and ferroptosis, whose breakdown promotes ferroptosis [78, 79].

**Fig. 4:**
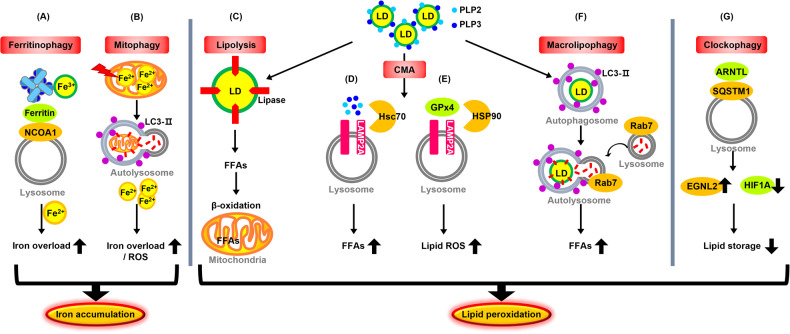
The role of autophagy in promoting ferroptosis. **A**, **B** NCOA4-mediated ferritinophagy (**A**) and Mitophagy (**B**) promote iron accumulation in ferroptosis. **C**–**G** Lipolysis mediated by lipases (**C**) and Hsc70/HSP90 chaperone-mediated autophagy (CMA) (**D**, **E**) and RAB7A-mediated macrolipophagy (**F**), and the cargo receptor SQSTM1-mediated clockophagy (**G**) promote lipid peroxidation in ferroptosis.

LD degradation is mediated by lipolysis, a process mediated by lipases, and a selective autophagic mechanism called lipophagy (Fig. 4) [80, 81]. Lipolysis enables the highly regulated release of FFAs from triacylglycerol (TAGs). FFAs are oxidized by the beta-oxidation pathway and converted to Acetyl-CoA [81]. Lipophagy is a process through the lysosomal pathway mediated by autophagy in two forms: chaperone-mediated autophagy (CMA) and macrolipophagy [77]. CMA is a lysosome-dependent protein degradation process, during which LD coat proteins, perilipin 2 (PLP2) and perilipin 3 (PLP3), bounded by cytosolic heat shock-associated protein 70 (Hsc70), are imported into the lysosome through a lysosome-associated membrane protein (LAMP2A) and are degraded [82]. As a general event during ferroptosis, GPX4, which can directly diminish lipid hydroperoxide production, is degraded via CMA. GPX4 degradation during ferroptosis is enhanced by HSP90, which can be activated under oxidative stress and increase LAMP2A stability [22]. Macrolipophagy mediated by Rab7 induces autophagic degradation of lipid droplets by LC3-II-positive membranes via lysosomal transportation and degradation. Rab7, used as a marker of the endolysosomal pathway, is required for the fusion of autophagosomes with lysosomes and promotes the decomposition of LDs. Lipophagy-mediated LDs degradation increases FFA production and promotes lipid peroxidation and subsequent ferroptosis [63, 83].

Cargo receptor SQSTM1-mediated clockophagy promotes lipid peroxidation during ferroptosis. Clockophagy is recently discovered as a type of autophagic degradation of aryl hydrocarbon receptor nuclear translocator-like (ARNTL) during ferroptosis, induced by type 2 ferroptosis inducers (e.g., RSL3 and FIN56) [22, 63]. Degradation of ARNTL increases the expression of EGLN2, inhibiting HIF1A (hypoxia inducible factor 1, alpha subunit) activation. HIF1A restricts ferroptosis by increasing fatty acid uptake and lipid storage, and reducing fatty acid β-oxidation, thus minimizing peroxidation-mediated cytomembrane damage. Consequently, downregulation of HIF1A reduces HIF1A-dependent accumulation of lipid droplets and promotes lipid peroxidation [84].

## Amplification of ferroptosis via autophagy and ROS feedback loop

There are evidences regarding feedback loop mechanisms in cancer that regulate autophagy and ferroptosis. These feedback loops involve signaling pathways that respond to changes in cellular metabolism and stress, and can either promote or inhibit autophagy and ferroptosis. One such feedback loop involves the p53 tumor suppressor protein, which is frequently mutated or lost in cancer cells. In normal cells, p53 can activate both autophagy and ferroptosis in response to cellular stress [85, 86]. However, in several cancer cells with p53 mutations, the loss of p53 function can lead to decreased autophagy and increased ferroptosis resistance. This can contribute to tumor growth and survival. Another feedback loop involves the NRF2-KEAP1 signaling pathway, which is involved in the regulation of oxidative stress and antioxidant responses in cells. In cancer cells, activation of the NRF2 pathway can lead to increased antioxidant defenses and resistance to ferroptosis [87]. However, NRF2 activation can also induce autophagy, which can promote ferroptosis in some contexts. In addition, other signaling pathways, such as the mTOR pathway and the unfolded protein response (UPR), can also regulate autophagy and ferroptosis in cancer cells through feedback mechanisms [88, 89]. These pathways respond to changes in nutrient availability, protein misfolding, and other stressors, and can either promote or inhibit autophagy and ferroptosis depending on the specific context. Collectively, the feedback loop mechanisms that regulate autophagy and ferroptosis in cancer cells are complex and context-dependent. Further research is needed to fully understand these mechanisms and their potential therapeutic implications for cancer treatment.

**Fig. 5:**
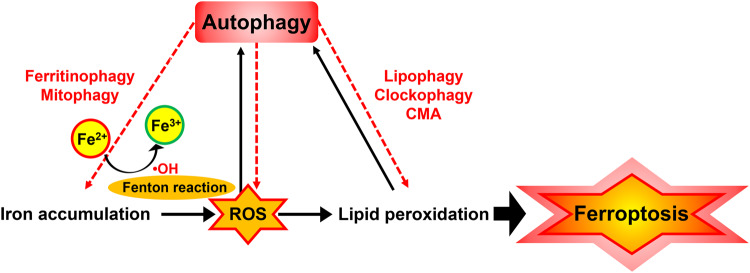
Autophagy as an enhancer of ferroptosis induced by iron accumulation-mediated ROS and lipid-based ROS. Autophagy is triggered by ROS in ferroptosis, caused by excess iron or lipid-dependent ROS accumulation, and then promotes iron and ROS accumulation, and this feedback loop contributes to the acceleration of ferroptosis. Ferroptosis is induced by increased ROS levels caused by elevated intracellular iron concentration, even without autophagy activation. In addition, autophagy alone without ROS generation could not induce ferroptosis. Thus, it cannot be concluded that ferroptosis is autophagic cell death and it is considered that autophagy enhances ferroptosis only under certain conditions.

The interrelationship between autophagy and ferroptosis has attracted increasing attention, providing a novel concept regarding cell death regulation [90]. Increasing evidence suggests that autophagy serves to ferroptotic cell death at least under certain conditions [22]. Here, we summarize the key perspectives on the impact of autophagy as an enhancer of ROS-dependent ferroptosis. Furthermore, autophagy is considered a reinforcer that promotes ROS-dependent ferroptosis as a synergistic effect via disruption of the redox balance. In ferroptosis, ROS triggers autophagy, resulting in ROS-mediated autophagy promoting ROS generation, and this feedback loop contributes to ferroptosis activation (Fig. 5).

### Activation loop of autophagy by ROS during ferroptosis

Classically, ferroptosis is an iron-dependent oxidative cell death caused by ROS and has been considered autophagy-dependent ferroptosis [22, 91]. However, in the absence of ROS generation, autophagy activation could inhibit chemical-induced ferroptosis in cancer cells. In these studies, autophagy activation was able to attenuate FIN56 or erastin-induced ferroptosis, even in the absence of ROS generation. The authors suggested that autophagy inhibited ferroptosis by reducing lipid peroxidation and iron accumulation [92–94].

In contrast, some additional studies explained the pathways and mechanisms accounting for ROS-initiated autophagy during ferroptosis [11, 20, 95]. A study investigated the role of ROS in regulating autophagy during ferroptosis [20]. They revealed that ROS generated during ferroptosis activated the AMPK pathway, which in turn phosphorylated and activated the ULK1 complex, a key initiator of autophagy. Another study investigated the role of the Nrf2 pathway in regulating autophagy during ferroptosis [11]. The authors showed that Nrf2, could also activate autophagy in response to ROS generated during ferroptosis. They suggested that Nrf2-mediated autophagy activation was a protective mechanism against ferroptosis-induced cell death. In addition, investigated the role of the MAPK pathway in regulating autophagy during ferroptosis [95]. They revealed that activation of the MAPK pathway by ROS generated during ferroptosis could activate the transcription factor TFEB, which in turn induced the expression of genes involved in lysosomal biogenesis and autophagy. These studies suggest that ROS generated during ferroptosis can activate various signaling pathways, such as AMPK, Nrf2, and MAPK, which in turn activate autophagy as a protective mechanism against ferroptosis-induced cell death.

Specifically, the activation of autophagy by ROS has been suggested in several studies. Iron accumulation-mediated ROS play an essential role in the induction of lipid peroxidation and activation of autophagy in ferroptosis. Liu et al. demonstrated that radiation-induced iron accumulation by the release of lysosomal iron generates ROS via the Fenton reaction and increases autophagy in a time-dependent manner [96]. In other studies, erastin, an inducer of ferroptosis, has been associated with the regulation of the antioxidant system through several molecules, including system Xc^-^ [97]. In this case, erastin-induced ROS triggers autophagy, and activated autophagy leads to iron-dependent ferroptosis by the degradation of ferritin and induction of transferrin receptor 1 (TfR1) expression [98, 99].

ROS-producing agents such as hydrogen peroxide and 2-methoxyestradiol (2-ME), cause autophagy and autophagic cell death. Blocking ROS generation through overexpression of MnSOD (manganese-superoxide dismutase) or using ROS scavengers also effectively blocked autophagy and ferroptosis [53, 91, 100]. In addition, acid sphingomyelinase (ASM), a key enzyme in sphingolipid metabolism, regulates autophagy by activating nicotinamide adenine dinucleotide phosphate oxidase (NADPH oxidase)-derived ROS in erastin-induced ferroptosis [101]. Beclin1, a critical protein associated with autophagy initiation, is upregulated by the ROS/p53 signaling pathway activated by ginsenosides, such as Rh4, during ferroptosis. Subsequently, the ROS scavenger N-acetyl-cysteine (NAC) reversed the inhibitory effect of Rh4 on cancer cell proliferation [102].

Subsequent accumulation of lipid-based ROS and loss of activity of the lipid repair enzyme, GPX4, induces lipid peroxidation and ferroptosis [103]. Previous studies have shown that lipid peroxidation can lead to autophagy activation through ROS induction. The products of lipid peroxidation activated by ROS induce autophagy [91, 104]. The signaling pathways of lipid peroxidation-driven autophagy are variable. Unsaturated lipid peroxidation-derived aldehydes such as 4-hydroxy-trans-2-nonenal (HNE) promote autophagy by a JNK-dependent mechanism [105, 106]. SIN-1-induced lipid peroxidation is associated with autophagy induction by TP53INP1, which interacts with LC3 and ATG8-family proteins [107, 108]. Lipid-soluble antioxidants such as resveratrol and vitamin E and the decrease in iron-mediated ROS via NAC and overexpression of superoxide dismutase 2 (SOD2) attenuated autophagy [96, 109, 110]. Therefore, autophagy is triggered by ROS, including iron accumulation-mediated and lipid-based ROS, which are the major causes of ferroptosis.

### Amplification loop of ROS by autophagy during ferroptosis

The autophagic mechanisms maintaining the balance between cell protection and cell death during ROS induction remain unclear [111]. Under normal environments, ROS-induced autophagy alleviates oxidative stress to protect cells from damage. For example, autophagy can protect cells by removing ROS to conserve mitochondrial integrity, avoid apoptosis, and promote antigen presentation. However, excessive ROS-induced autophagy can cause autophagic cell death and amplify ferroptosis by promoting higher ROS levels under certain circumstances [91, 100].

Unregulated iron homeostasis can result in excessive iron accumulation and subsequently lead to damage to proteins, lipids, and DNA through the generation of free radicals and oxidative stress [112]. Several studies have demonstrated that ferritinophagy, a type of selective autophagy, contributes to ferroptosis by inducing iron-dependent ROS production by mediating the degradation of ferritin. Ferritin degradation eventually results in the cytosolic release of chelated iron, thereby inducing oxidative stress [20, 113]. Knockout or knockdown of ATG5 and ATG7 inhibited ferroptosis induced by erastin with diminished intracellular Fe^2+^ levels, and lipid peroxidation [20, 51]. A dithiocarbamate derivative, 2-pyridylhydrazone dithiocarbamate s-acetic acid (PdtaA), decreased GSH and increased lipid peroxidation and ferritinophagy-mediated ROS production. Autophagy inhibition using the autophagy inhibitor 3-methyladenin (3-MA) counteracted the effect of PdtaA on ferritinophagy and ferroptosis induction via downregulation of GPx4 and xCT [113].

The relationship between autophagy and ferroptosis has a complicated interaction during oxidative stress. Several studies have demonstrated that ROS-mediated autophagy promotes ROS-induced lipid peroxidation and cell death [91]. Blocking ROS-induced autophagosome accumulation through the autophagy inhibitor 3-MA or knocking down autophagy genes, including beclin-1, ATG-5, and ATG-7, effectively blocked ROS generation and oxidative stress-induced cell death [53, 100]. Autophagy mediated by ASM, a key enzyme in sphingolipid metabolism, plays a critical role in GPX4 degradation via ferroptosis inducers and ferroptosis activation. During the activation of ASM by erastin, autophagy inhibitors such as Bafilomycin A1, hydroxychloroquine (HCQ), and ammonium chloride (NH4Cl) inhibit erastin-induced ROS generation and ferroptosis [101]. Furthermore, autophagy induction following GSH depletion plays a key role in the increase of oxidative stress and lipid peroxidation in ferroptosis by GSH depletion [98].

Some additional studies could explain the pathways and mechanisms accounting for autophagy-dependent generation of ROS during ferroptosis. Yang and colleagues investigated the role of the autophagy protein p62/SQSTM1 in regulating ROS generation during ferroptosis. The authors showed that p62/SQSTM1 could bind to and stabilize the ferroptosis regulator NCOA4, which in turn promoted the transfer of iron from ferritin to the lipid peroxidation system, generating ROS and promoting ferroptosis. The authors suggested that p62/SQSTM1-mediated ROS generation was a positive feedback loop that amplified ferroptosis [114]. Another study investigated the role of the autophagy protein ATG5 in regulating ROS generation during ferroptosis. ATG5-deficient cells had reduced levels of ROS and were resistant to ferroptosis, while ATG5-overexpressing cells had increased levels of ROS and were more susceptible to ferroptosis. The authors suggested that ATG5-mediated ROS generation was a positive feedback loop that amplified ferroptosis [51]. In addition, the role of autophagy in regulating ROS generation during ferroptosis induced by the compound FIN56. Autophagy activation increased ROS levels and sensitized cancer cells to FIN56-induced ferroptosis, while autophagy inhibition reduced ROS levels and protected cells from ferroptosis. The authors suggested that autophagy-mediated ROS generation was a positive feedback loop that amplified ferroptosis [93]. These studies suggest that autophagy can amplify ROS generation during ferroptosis by regulating key pathways involved in iron metabolism and lipid peroxidation, such as the p62/SQSTM1-NCOA4 axis and ATG5-mediated signaling.

Therefore, excessive autophagy stimulated by oxidized lipids and prolonged iron-mediated ROS plays a critical enhancer role in ferroptosis via the promotion of oxidative stress through enhancement of iron accumulation by ferritinophagy and lipid peroxidation by lipophagy, clockophagy, and CMA [22, 115]. Thus, even if autophagy alone does not lead to ferroptosis, it may act as an amplifier during ferroptosis, and we can conclude that ferroptosis is autophagic cell death under specific conditions.

## Perspectives

Ferroptosis is a cell death process driven by iron-dependent lipid peroxidation [41, 42]. Recently, studies have expanded on the molecular mechanisms underlying ferroptosis and revealed a complex regulatory mechanism [22]. Ferroptosis has been shown to play an important regulatory role in the occurrence and development of several diseases [2, 116]. Direct control of ferroptosis via modulation of lipid peroxidation influences disease therapy, however, the sources of ferroptosis induction are diverse and ferroptosis is also implicated in disease development. Highly metastatic and resistant cancers are extremely vulnerable to ferroptosis inducing treatment; however, the pathophysiology of neurological diseases including neurodegeneration, stroke, and neurotrauma, are associated with ferroptosis [50, 117]. Therefore, elucidating the mechanisms underlying ferroptosis can improve our understanding of human diseases and provide potential prevention and treatment interventions for various diseases [117].

In this review, we discussed the role of autophagy as a ferroptosis enhancer via the ROS amplification loop. We summarized that autophagy is triggered by ROS including iron accumulation-mediated ROS and lipid-based ROS in ROS-dependent ferroptosis and then acts to amplify iron accumulation and lipid peroxidation and subsequently can induce autophagic cell death under specific conditions. Accordingly, therapeutic strategies targeting the crosstalk between autophagy, iron, and ROS in ferroptosis could provide novel promising directions for the treatment of diseases and improve therapeutic options [93]. In addition, various types of autophagy such as ferritinophagy, mitophagy, lipophagy, clockophagy, contribute to ferroptosis. However, the type of autophagy that contributes to ferroptosis remains unclear, and further studies are needed to identify novel target molecules that regulate autophagy-dependent ferroptosis. Therefore, additional research on the relationship between autophagy and ferroptosis mechanisms in pathological pathways can provide insights into disease development. This review would pave the way for understanding the role of autophagy in the connection between oxidative stress, autophagy, and ferroptosis.
